# Considerations of growth factor and material use in bone tissue engineering using biodegradable scaffolds in vitro and in vivo

**DOI:** 10.1038/s41598-024-75198-3

**Published:** 2024-10-28

**Authors:** Karen M. Marshall, Jonathan P. Wojciechowski, Vineetha Jayawarna, Abshar Hasan, Cécile Echalier, Øystein Øvrebø, Tao Yang, Kun Zhou, Janos M. Kanczler, Alvaro Mata, Manuel Salmeron-Sanchez, Molly M. Stevens, Richard O. C. Oreffo

**Affiliations:** 1 Bone and Joint Research Group, Centre for Human Development, Stem Cells and Regeneration, Institute of Developmental Sciences, University of Southampton, Southampton, SO16 6YD United Kingdom; 2 Department of Materials, Department of Bioengineering and Institute for Biomedical Engineering, Imperial College London, London, SW7 2AZ United Kingdom; 3 School of Engineering, Centre for the Cellular Microenvironment, Advanced Research Centre, University of Glasgow, Glasgow, G11 6EW United Kingdom; 4 School of Pharmacy, University of Nottingham, Nottingham, NG7 2RD United Kingdom; 5 Department of Chemical and Environmental Engineering and NIHR Nottingham Biomedical Research Centre, University of Nottingham, Nottingham, NG7 2RD United Kingdom; 6 Kavli Institute for Nanoscience Discovery, Department of Physiology, Anatomy and Genetics, Department of Engineering Science, University of Oxford, Oxford, OX1 3QU, United Kingdom

**Keywords:** Bioactive coating, Biomaterial, Bone tissue engineering, CAM assay, Animal models, Preclinical research, Stem-cell differentiation, Stem-cell research, Fracture repair

## Abstract

Bone tissue engineering aims to harness materials to develop functional bone tissue to heal ‘critical-sized’ bone defects. This study examined a robust, coated poly(caprolactone) trimethacrylate (PCL-TMA) 3D-printable scaffold designed to augment bone formation. Following optimisation of the coatings, three bioactive coatings were examined, i) elastin-like polypeptide (ELP), ii) poly(ethyl acrylate) (PEA), fibronectin (FN) and bone morphogenetic protein-2 (BMP-2) applied sequentially (PEA/FN/BMP-2) and iii) both ELP and PEA/FN/BMP-2 coatings applied concurrently. The scaffold material was robust and showed biodegradability. The coatings demonstrated a significant (*p* < 0.05) osteogenic response in vitro in alkaline phosphatase gene upregulation and alkaline phosphatase production. The PCL-TMA scaffold and coatings supported angiogenesis and displayed excellent biocompatibility following evaluation on the chorioallantoic membrane assay. No significant (*p* < 0.05) heterotopic bone formed on the scaffolds within a murine subcutaneous implantation model, compared to the positive control of BMP-2 loaded collagen sponge following examination by micro-computed tomography or histology. The current studies demonstrate a range of innovative coated scaffold constructs with in vitro efficacy and clearly illustrate the importance of an appropriate in vivo environment to validate in vitro functionality prior to scale up and preclinical application.

**Supplementary Information:**

The online version contains supplementary material available at 10.1038/s41598-024-75198-3.

## Introduction

Advances in healthcare have contributed to a global increase in population aging. However, this welcome increase in life expectancy, results in major challenges with the inability to effectively regenerate tissues, resulting in chronic illness, disability and rising healthcare expenses. The field of bone tissue engineering seeks to repair or regenerate bone tissue, harnessing innovative materials that can replace autografts (i.e. the patient’s own bone), allografts (e.g. decellularized donor human bone), or a plethora of calcium phosphate-based materials currently used in clinical settings. Defects arising in bone may be due to trauma, non-union, infection, oncology treatment or congenital conditions^1^. Amputation may be required following substantial soft tissue, vascular and/or nerve damage making the limb unsalvageable^2^. Autograft remains the gold standard material for bone defect repair, as an autograft is non-immunogenic, osteoinductive (due to the presence of growth factors), osteoconductive as bone is a 3D scaffold and osteogenic given the cellular component^3–5^. The limitations to autograft application are however, donor site complications such as infection, pain and the limited volume of graft material available^6^. Thus, the ideal bone substitute material is biocompatible, bioresorbable, osteoconductive, osteoinductive, porous and with a comparable structure and strength to bone, while being easy to apply and cost effective^1^.

In our previous work, we designed non-degradable ‘octet-truss’ polyamide (nylon) and titanium scaffolds via SLS 3D printing to have apparent moduli within the ranges of trabecular bone (i.e. 7100 MPa = ten-fold higher, 1400 MPa = matched, 220 MPa = matched to lower range) as synthetic bone grafts. In the study*,* strongly- and weakly-responding animals were identified, in which the more compliant scaffolds (*i.e.,* apparent modulus = 220 MPa) showed enhanced bone formation in a sheep femoral condyle defect model that was inversely proportional to stiffness, and strain-driven for strongly-responding animals^7^. The limitations of the study were in the use of a non-degradable scaffold which could not be removed after regeneration of the bone tissue and the absence of any bioactive coatings to further promote the bone healing process.

To address these challenges, the current work details examination of a PCL trimethacrylate (PCL-TMA) material as a biocompatible, biodegradable, 3D-printable polymer scaffold to fulfil some of the ideal properties of a bone substitute material^8–10^. Polyester based materials are commonly used as degradable materials in medical devices^11^. PCL-TMA was chosen as a candidate biodegradable material because of its mechanical and material properties. Considering the mechanical properties of PCL-TMA at a high degree of functionalisation (i.e., > 95%), the Young’s modulus is comparable to the lower range of cancellous bone, the PCL-TMA materials can handle stresses near the upper range of cancellous bone, and the strain is approximately two-fold higher than cancellous bone^12^. In addition, the degree of functionalisation and low molecular weight of the PCL-TMA material provides low viscosity and fast-curing kinetics which are favourable for SLA based 3D printing.

To confer a novel biodegradable scaffold material with osteogenic properties, bioactive surface coatings for application in large bone defects were examined in vitro and in vivo with potential clinical translation on the PCL-TMA octet-truss scaffold. Three bioactive coatings were examined: i) elastin-like polypeptide (ELP), ii) poly (ethyl acrylate) (PEA), fibronectin (FN) and bone morphogenetic protein-2 (BMP-2) applied sequentially (PEA/FN/BMP-2) or iii) ELP and PEA/FN/BMP-2 applied concurrently on the scaffold surface.

Elastin-like polypeptides (ELPs) are biocompatible, artificial, elastin-based polypeptides , not recognised as foreign by the immune system^13^. Mineralised ELP coated scaffolds provide a novel coating based on the interaction between organic matter and inorganic crystal formation, with the formation of fluorapatite spherulites. These structures develop as prism-like microstructures and mature into macroscopic circular structures, which can encapsulate large and uneven material surfaces^14^. ELP coatings were selected given their capacity to coat implants^15^ as well as their potential to mineralize while recreating the stiffness of hard tissues^16^ and enhance osseointegration in bone tissue engineering^17^.

Previous work from the Salmeron-Sanchez group has demonstrated the PEA/FN/BMP-2 coating as an osteoinductive and osteoconductive material for bone regeneration^18^. PEA is non-biodegradable; however, PEA may be metabolised following application as a thin layer (< 10s of nm)^18^. Fibronectin forms organised fibrils as it unfolds when in contact with PEA, enabling cell interaction with the FN adhering and aligning on the material^19^. Previous work has shown FN can bind to BMP-2, presenting BMP-2 to skeletal cells and facilitating osteogenic differentiation in vitro^20^*.* Subsequently, a mouse radial defect study illustrated enhanced bone repair with a polyimide ‘sleeve’ coated in PEA/FN/BMP-2 compared to control ‘sleeves’^20^. The clinical use of this FN and BMP-2 growth factor interaction on PEA has been validated in veterinary cases of complicated, non-healing fractures^18,21^. Furthermore, in comparison to other clinical reports using 0.5 mg/mL of BMP-2 solution for non-union cases, this method of binding BMP-2 to PEA/FN utilises lower concentrations of BMP-2^22^.

Our aim was to create a biodegradable, biocompatible, osteogenic scaffold which could be used to repair lower limb bone defects. The objectives were to determine the cytocompatibility, biocompatibility and osteogenic properties of biodegradable coated scaffolds. The hypotheses under examination were specifically: i) the scaffold material and coatings would be biocompatible, ii) coated scaffolds would stimulate greater bone formation around the scaffold in vivo compared to uncoated scaffolds, iii) the ELP/PEA/FN/BMP-2 coated scaffold would create significantly greater bone volume than the ELP alone or PEA/FN/BMP-2 coated scaffolds.

The cytocompatibility of the PCL-TMA and the coatings were examined using alamarBlue™ HS cell viability reagent. The efficacy of the coatings to induce osteogenic differentiation was evaluated by ALP specific activity analysis as well as measuring osteogenic gene expression of human bone marrow stromal cells (HBMSCs). Subsequently, the biocompatibility, ability to integrate with vascularised tissue and to support angiogenesis was examined using the chorioallantoic membrane (CAM) assay. Finally, the ability of the selected bioactive scaffold constructs to induce bone formation in a heterotopic site was investigated, using the murine subcutaneous implantation model, with micro-computed tomography (µCT) quantification and histological analysis. This study illustrates the optimisation and critical analysis of in vitro and in vivo findings required on the path to clinical translation of innovative bioactive coatings.

## Materials and methods

### Materials

Reagents were purchased as follows: ethyl acrylate (Sigma, UK), ELP with statherin sequence (SN_A_15) (Technical Proteins Nanobiotechnology, Valladolid, Spain); collagenase (Gibco, UK); human fibronectin and human recombinant BMP-2 (R&D systems, Biotechne, UK); recombinant human BMP-2 (Infuse/InductOS® Bone graft kit, Medtronic, USA); alcian blue 8X, light green SF, orange G 85% pure, paraformaldehyde 96% extra pure, phosphomolybdic acid hydrate 80% (Acros Organics); Picrosirius Red, Van Gieson’s stain, Weigert’s Haematoxylin Parts 1 and 2 (Clintech Ltd, UK); benzoyl peroxide, GMA solution B, JB4 solution A (Polysciences); GoTaq qPCR master mix, Herring sperm DNA, RNeasy mini prep RNA extraction kit (Promega); phosphate buffered saline (PBS), trypsin/ethylenediaminetetraacetic acid (EDTA), Dulbecco’s Modified Eagle Medium (DMEM), Alpha Minimum Essential Medium (αMEM), penicillin–streptomycin (Scientific Laboratory Supplies, SLS); 4-nitrophenol solution 10 nM, acetic acid, acetone, acid fushsin, alizarin red S, alkaline buffer solution, ascorbic acid-2-phosphate, beta-glycerophosphate disodium hydrate salt (βGP), cell lytic M, dexamethasone, fast violet B salts, glycine, histowax, hydrochloric acid, iodoacetamide, ipegal, L-glutamic acid, Naphthol AS-MX phosphate 0.25%, parafilm, PBS (with CaCl_2_/MgCl_2_), phenyl methyl sulphonyl fluoride, phosphatase substrate, polysorbate 80, ponceau xylidine, silver nitrate, sodium chloride, sodium hydroxide pellets, sucrose, TRIS–EDTA (TE) buffer solution (Merck, UK); Embedding capsule (TAAB Laboratories equipment); alamarBlue™ HS Cell Viability Reagent, 70 µM cell strainer, dibutyl phthalate xylene (DPX), ethidium homodimer-1, fetal calf serum (FCS), fisherbrand grade 01 cellulose general purpose filter paper, Histoclear, isopropanol, methyl benzoate, Quanti-IT™ Picogreen™ ds DNA reagent, Taqman® Reverse Transcription Kit, Vybrant™ CFDA SE Cell Tracer Kit (Thermofisher Scientific, UK); Fast green and sodium thiosulphate (VWR); Lubrithal (Dechra, UK), Isoflurane (Dechra, UK), Buprenorphine (Buprecare® multidose, Animalcare, UK) and Vetasept® sourced from MWI animal health, UK. Uncoated vacutainers, 3-way stopcock and 5/0 PDS II suture (Ethicon, USA) from NHS supply chain. All other consumables and reagents were from Sigma-Aldrich, UK.

### Production of PCL trimethacrylate scaffold material

PCL-trimethacrylate of this molecular weight has been synthesised and 3D printed via stereolithography (SLA) previously^8–10^. Silica gel (40–63 μm; VWR chemicals) was used as a stationary phase. ^1^H NMR and ^13^C NMR spectra were recorded on a JEOL 400 NMR spectrometer, with working frequencies of 400 MHz for ^1^H nuclei.

#### Poly(caprolactone) trimethacrylate synthesis

Poly(caprolactone) triol, M_n_ = 300 Da, (50 g, 0.17 mmol, 1 eq), anhydrous dichloromethane (350 mL) and triethylamine (100 mL, 0.72 mmol, 4.3 eq) were added to a 1 L two-necked round bottom flask. The reaction was placed under a nitrogen atmosphere and then cooled in an ice-water bath for 15 min. A pressure-equalising dropper funnel charged with methacryloyl chloride (65 mL, 0.67 mmol, 4 eq) was attached to the round bottom flask. The methacryloyl chloride was added dropwise over approximately 3 h. The reaction was covered with aluminium foil to protect it from light and allowed to stir and warm to room temperature (RT) overnight. The next day, methanol (50 mL) was added to quench the reaction, which was allowed to stir at RT for 30 min. The reaction mixture was transferred to a separating funnel and washed with 1 M aqueous hydrochloric acid solution (5 × 250 mL), saturated sodium bicarbonate solution (1 × 250 mL) and brine (1 × 250 mL). The organic layer was then dried with anhydrous magnesium sulphate, filtered and concentrated via rotary evaporation. The crude yellow liquid was then purified using a silica plug, with dichloromethane as the eluent. Fractions containing PCL-trimethacrylate were pooled and concentrated via rotary evaporation. The PCL-trimethacrylate was transferred to a brown glass vial and dried using a stream of air (through a plug of CaCl_2_) overnight to yield the title compound as a slightly yellow viscous liquid (82.2683 g). The PCL-trimethacrylate was supplemented with 200 ppm (w/w) of 4-methoxyphenol (MEHQ) as an inhibitor (16.34 mg).

^1^H NMR (400 MHz, CDCl_3_) δ 6.14 – 6.04 (m, 3H), 5.63 – 5.50 (m, 3H), 4.18 – 4.00 (m, 9H), 2.36–2.32 (m, 3H), 1.94 (m, 9H), 1.75 – 1.47 (m, 9H), 1.47 – 1.32 (m, 2H), 1.02 – 0.83 (m, 3H).

The characterisation data agrees well with that previously reported, however, with an improved degree of functionalisation (> 95%).

#### 3D Printing of PCL-trimethacrylate

PCL-trimethacrylate octet-truss scaffolds were designed based on a modified body centre cubic unit cell (diameter = 5 mm, and height = 5 mm, strut diameter = 0.5 mm, surface area 143.4 mm^2^), denoted PCL-TMA scaffolds. This scaffold design was chosen to mimic the unit cell geometry of larger octet-truss style scaffolds as previously reported by Reznikov et al. but in a format suitable for in vitro studies^7^. The PCL-TMA scaffolds were printed using masked (SLA) 3D printing on a Prusa SL1 or SL1S. The resin was prepared for 3D printing by first dissolving 0.1% (w/w) 2,5-thiophenediylbis(5-tert-butyl-1,3-benzoxazole) (OB +) as a photoabsorber in the PCL-TMA by stirring at RT for 1 h. Finally, 1.0% (w/w) diphenyl(2,4,6-trimethylbenzoyl)phosphine oxide (TPO-L) as a photoinitiator was added to the resin.

After printing, the scaffolds were rinsed with ethanol and removed from the build plate. Scaffolds were sonicated in ethanol (5 × 5 min) and allowed to dry for 15 min at RT. The scaffolds were post-cured using a Formlabs Form Cure for 60 min at RT. After post-curing, the scaffolds were soaked into ethanol overnight at RT on a rocker (100 rpm), rinsed with ethanol (3x) and allowed to dry at RT before being ELP and/or PEA coated and EO sterilised, or left uncoated and EO sterilised.

#### Scanning *Electron* Microscopy (SEM)

The PCL-TMA unit cell structures were imaged using a Zeiss Leo Gemini Scanning Electron Microscope fitted with an SESI detector. The samples were coated with a 15 nm chromium coating prior to imaging. All images were taken using 8 kV accelerating voltage.

#### ATR-FTIR

The PCL-TMA unit cells were measured using ATR-FTIR using a Bruker Alpha II Compact FTIR. A background scan of 128 scans was used. Samples were measured using 128 scans at a resolution of 1 cm^−1^. The FTIR spectra was smoothed using a Whittaker-Hayes smoothing function, and baseline corrected using an adaptive smoothness parameter penalized least squares method (asPLS).

#### Compressive testing

The compressive mechanical properties of the scaffolds were measured using a Bose Electroforce TA 3200 equipped with uniaxial load cells. Due to the geometry of the unit cell scaffolds, the effective elastic modulus was measured. A 1 N preload was implemented, with the effective elastic modulus of the PCL-TMA unit cells measured between 1 – 3% strain (n = 5).

#### Accelerated degradation

To confirm the biodegradation of PCL-TMA an accelerated degradation study using sodium hydroxide was conducted. The unit cells were first washed five times in milli Q water, then twice in absolute ethanol, wiped off with Kimtech precision wipes, then dried in a vacuum desiccator overnight, before the initial weight (m_0_) of the cell cylinder was measured. The cell cylinders were then placed in individual 1.5 mL Eppendorf tubes, before 1 mL of 2 M sodium hydroxide was added. The samples were then incubated at 37 °C for 1, 2, 4, 7, 14, or 21 days before they were taken out, washed and dried in a similar manner as described above, then the new weight (m_1_) was measured. Three repeats were conducted at each time point.

### Coating of the scaffold material

The scaffold coating process and work flow used in this in the study are illustrated in Supplementary Figure 1.

#### PEA/FN/BMP-2 coating of the scaffold material

PEA coating of materials was performed as previously described^18^. Briefly, the scaffolds were treated in air plasma for 5 min before being exposed to monomer plasma. Plasma polymerization was carried out in a custom-made capacitively coupled plasma installation for low-pressure plasma in a 15-L T-shaped reactor made of borosilicate glass and stainless-steel end plates sealed with Viton O-rings. A vacuum was produced by a rotary pump or a scroll pump (both BOC Edwards, UK), with working pressures for the monomer plasma from 0.15 to 0.25 mbar. The plasma was initiated via two capacitively coupled copper band ring electrodes located outside of the reactor chamber and connected to a radiofrequency generator (Coaxial Power System Ltd.). The monomer pressure was controlled via speedivalves (BOC Edwards, UK) and monitored with a Pirani gauge (Kurt J. Lesker). PEA was applied on the material surfaces for 15 min at a total radiofrequency power of 50 W. The samples were sterilized afterwards using ethylene oxide (EO).

The application of the BMP-2 coating was optimised by investigating different diluents and BMP-2 manufacturers to ensure the biological activity of the BMP-2 protein (Supplementary Table 1, Supplementary Figures 2, 3 and 4). For experiments with the PEA/FN/BMP-2, or ELP/PEA/FN/BMP-2 coated PCL-TMA scaffolds, FN and InductOs® BMP-2 were diluted in PBS with calcium chloride (CaCl_2_) and magnesium chloride (MgCl_2_) added. A maximum of three scaffolds were placed into a non-coated 11 mL vacutainer and 1 mL of FN solution (20 µg/mL) was added. A vacuum was created and after 1 h in the sterile hood at RT, the FN solution was removed. PBS (containing CaCl_2_/MgCl_2_) was added at 1–2 mL per vacutainer to rinse off non-bound FN and repeated once. The scaffolds were handled with sterile forceps and transferred to new vacutainers. 1 mL of BMP-2 solution (100 ng/mL for in vitro experiments or 5 µg/mL for CAM assay and subcutaneous implantation experiments) was added to each tube. The formation of a vacuum was repeated. After 1 h at RT, the BMP-2 solution was removed and the scaffolds rinsed twice in PBS (containing CaCl_2_/MgCl_2_) prior to use.

#### ELP coating of the scaffold material

ELP coating of scaffolds was based on previous published methods^16^. In brief, lyophilized ELP powder was dissolved in solvent mixture of DMF/DMSO (at 9/1 ratio) to prepare 5% (w/v) ELP solution followed by addition of hexamethyl diisocyanate (HDI) crosslinker (cross-linker to lysine ratios of 12/1). 3D printed polyamide or PCL-TMA scaffolds were immersed in the ELP solution for 10–15 s and left to dry overnight at RT (22 °C) inside a glovebox (BELLE Technology, UK) maintained at a humidity < 20%. Dry ELP coated scaffolds were washed several times with deionised water (dH_2_O) to remove excess HDI and were termed as ELP coated scaffolds^17^.

The ELP coating was initially exogenously mineralised in a fluorapatite solution, however, this was found to affect the material property of the PCL-TMA. Therefore, ELP coating without exogenous mineralisation was deemed a suitable coating material due to results confirming that the ELP coating could be mineralised in vitro using surrounding mineralising media components (Supplementary information).

For the ELP/PEA/FN/BMP-2 coated scaffolds, the ELP coating was applied, followed by PEA and subsequent EO sterilisation. Finally, the FN and BMP-2 were adhered to the scaffolds as described in section "PEA/FN/BMP-2 coating of the scaffold material".

### HBMSC isolation and culture

#### Isolation and culture of HBMSCs

Human bone marrow samples were collected from patients undergoing hip replacement surgery, with prior informed consent from the patients. The methods were performed in accordance with the relevant guidelines and regulations at the University of Southampton. The samples were identified only by sex (male (M) or Female (F)) and age (e.g., F60) to maintain confidentiality, with approval of the University of Southampton’s Ethics and Research Governance Office and the North West-Greater Manchester East Research Ethics Committee (18/NW/0231) for use of the tissue for research. In a class II hood, under sterile conditions, 5–10 mL alpha-Minimum Essential Medium (α-MEM, Lonza, UK) or Dulbecco’s Modified Eagle Medium (DMEM, Lonza, UK) was added to the universal tube of marrow and shaken vigorously to extract the HBMSCs. A 3 mL sterile Pasteur pipette was used to remove the supernatant media/cellular debris mix to a 50 mL falcon tube and washing was repeated until the bone was light pink/white in colour. The cell suspension was centrifuged (272 g Heraeus mega 1.0R centrifuge) for 5 min. The supernatant was removed, the pellet resuspended in α-MEM or DMEM and passed through a 70 µM cell strainer (Fisher Scientific, UK) to remove bone and fat debris. The suspension was centrifuged and the supernatant poured off. The pellet was resuspended in basal media (α-MEM, 10% fetal calf serum (FCS), 1% penicillin–streptomycin (P/S)) and the HBMSCs were cultured in T175 flasks at 37 °C in 5% CO_2_/balanced air until approximately 80% confluent. Collagenase (2% solution and or 0.22 IU/mg) was used prior to trypsin solution (1 × concentration (Stock Trypsin/EDTA (10X), includes 1,700,000 U\L trypsin 1:250 and 2 g/L Versene® (EDTA)), for passaging and seeding onto scaffolds.

Osteogenic media consisted of α-MEM, 10% FCS, 1% P/S, ascorbate-2-phosphate 50 mM (2 µL/mL), dexamethasone 10 μM (1 µL/mL). All media was changed every 3–4 days.

#### Cell seeding onto scaffolds

2.5 × 10^4^ Passage 2 (P2) HBMSCs (F79) per scaffold were used for cell viability/alamarBlue™ HS experiments and 5 × 10^4^ Passage 1 (P1) HBMSCs were used for biochemistry (F75) and molecular (M59) experiments (Supplementary Figure 5). Each PCL-TMA octet-truss scaffold was added individually to a 2 mL Eppendorf tube and 500 µL cell suspension (α-MEM, 1% P/S, without FCS) was added. The Eppendorf tubes were placed in a 50 mL falcon tube (6 per tube) for positioning horizontally on the MACSmix™ Tube Rotator enabling a maximum of 24 scaffolds to be seeded per rotator machine. The Eppendorf tubes were fully sealed and therefore the air available to cells was that only within the tubes. The media maintained the normal pink colour throughout cell seeding, indicating the pH of the media was unaltered. FCS was added at approximately 10% (55 µL) to each tube after 3 h. After 24 h, each scaffold was moved to individual wells of a 24 well plate containing 1.5 mL basal or osteogenic media. Culture was performed at 37 °C in 5% CO_2_/balanced air, with media changed every 3 days. Each scaffold coating type/media condition was set up in triplicate.

### Assessment of cytocompatibility of PCL-TMA and coatings

#### alamarBlue™ HS Cell Viability assay

For cell viability experiments alamarBlue™ HS Cell Viability Reagent was added to basal media at 10% (v/v) concentration. Fluorescence measurements were taken at day 1 (when the PCL-TMA scaffolds were removed from the Eppendorf tube after 24 h of cell seeding) and on day 14 (each PCL-TMA scaffold was moved to a new 24 well plate to ensure only the cells adhered to the scaffold were quantified). A 1 mL aliquot of media/alamarBlue™ HS mix was added to each well containing a scaffold and to 3 wells with no scaffold as background measurements and incubated for 4 h at 37 °C in 5% CO_2_/balanced air. After 4 h, 100 µL samples were taken from each well and plated in triplicate in a black 96 well plate. The fluorescence was measured using the GloMax® Discover Microplate Reader (Promega, UK) at Green 520 nm excitation and 580–640 nm emission and the average background measurement was subtracted from each sample well.

#### Labelling of live and dead HBMSCs

Vybrant™ CFDA SE Cell Tracer 10 μM and 5 µg/mL Ethidium homodimer-1 in PBS, were used to label live and dead cells respectively on PCL-TMA scaffolds at day 1 and day 14 post alamarBlue™ HS analysis. Media/alamarBlue™ HS was removed and scaffolds washed twice in PBS. A 1 mL aliquot of labelling solution was added to cover the scaffolds/cells and incubated for 15 min at 37 °C in 5% CO_2_/balanced air. The labelling solution was removed and replaced with α-MEM/1% P/S/10% FCS and culture continued for 30 min. The samples were imaged under fluorescence microscopy using the FITC filter (excitation 485 nm, emission 515 nm) for live cells and RHODA/TRITC filter (excitation 510–560 nm, emission 590 nm) for dead cells, with a Zeiss Axiovert 200 microscope and Axiovision 4.2 imaging software.

### Biochemistry assays for HBMSC differentiation analysis

#### Alkaline phosphatase specific activity measurement

Placement of scaffolds in basal or osteogenic media after 24 h of cell seeding was determined as day 0. Therefore, day 1 was after 24 h of culture in basal or osteogenic media and so on until the day 7 when the HBMSCs were lysed. HBMSCs attached to PCL-TMA scaffolds were washed twice in PBS and the scaffolds were transferred to individual 2 mL Eppendorf tubes. A 200 µL aliquot of Cell lytic M was added to cover the scaffold and left in contact for 15 min at RT with vortexing performed three times every 5 min. The Eppendorf tubes were stored at −80 °C.

ALP activity was measured using a colourimetric absorption assay. P-nitrophenol phosphate (pNPP) production was measured against standards. A 10 µL aliquot of cell lysate was transferred to a clear 96 well plate and 90 µL of substrate was added. The plate was incubated at 37 °C until a yellow colour change was noted and the time recorded for this change. A 100 µL aliquot of 1M sodium hydroxide solution was added to stop the reaction, prior to reading absorbance on the GloMax® Discover (spectrophotometer) at 405 nm. The same centrifuged cell lysate samples were used as for ALP quantification. PicoGreen™ was diluted 1/200 in TE (1x) buffer and added to all wells, including standards. The plate was left on the benchtop at RT for 5 min prior to the quantity of DNA being measured using a fluorescence assay at blue 475 nm excitation and 500–550 nm emission, in a GloMax® Discover Microplate Reader (Promega, UK). Results expressed as nanogram (ng) of DNA. ALP specific activity was calculated as ALP produced per ng of DNA as nmol pNPP/ng DNA/hr.

### Osteogenic gene expression analysis

#### RNA extraction

RNA extraction was performed using the Promega ReliaPrep™ RNA Cell Miniprep System instructions. Scaffolds were washed in PBS and transferred to 2 mL Eppendorf tubes with BL-TG buffer (250 µL) for 15 min to lyse the cells prior to storage at -80 °C. The tubes were thawed and isopropanol (85 µL) added, prior to brief vortexing. The lysate was transferred to a minicolumn within a collection tube. The column was washed in a series of steps as per the kit instructions and the final RNA eluted with nanopure water (15 µL). RNA quantification and purity measurement were performed using the Nanodrop 2000 spectrophotometer.

#### Reverse transcription

RNA quantity for conversion to cDNA was standardised for all samples with dilution in nanopure water to a volume of 9.6 µL in a 0.2 mL PCR reaction tube. The reagents of the TaqMan® Reverse Transcription (RT) Kit were mixed in quantities instructed to make RT master mix and 10.4 µL was added to water/RNA mix to give a final 20 µL reaction volume. The tubes were placed in a SimpliAmp thermal cycler (Applied Biosystems, UK) set to 10 min at 25 °C for primer incubation, 30 min at 37 °C for reverse transcription and 5 min at 95 °C for the reverse transcription inactivation and 4 °C to hold samples until retrieval for storage at −20 °C.

#### Quantitative Polymerase Chain Reaction (qPCR)

Quantitative qPCR was performed using GoTaq PCR master mix. The master solution was made from 10 µL GoTaq PCR master mix, 0.75 µL of forward primer, 0.75 µL of reverse primer and 7.5 µL ultrapure water (Human β-actin gene forward sequence 5’-3’ GGCATCCTCACCCTGAAGTA, reverse sequence AGGTGTGGTGCCAGATTTTC, Human *ALP* gene forward sequence 5’-3’ GGAACTCCTGACCCTTGACC, reverse sequence 5’-3’ TCCTGTTCAGCTCGTACTGC and Human *Collagen1A1* gene forward sequence 5’-3’ CCCTGGAAAGAATGGAGATGAT and reverse sequence 5’-3’ ACTGAAACCTCTGTGTCCCTTCA). The final 20 µL reaction volume was made of 2 µL of cDNA sample and 18 µL of master solution in a 96 well PCR plate, sealed and centrifuged briefly before analysis using 7500 Real Time PCR system (Applied Biosystems, UK). The resulting data were collected using the AB7500 SDS Software (Applied Biosystems, UK). Ct values for each sample were normalised to the housekeeping gene β-actin, after optimisation experiments revealed β-actin to be a reliable housekeeping gene and there was often not sufficient RNA yield to allow multiple housekeeping genes to be used. Relative-expression levels of each target gene were calculated using the ∆∆ C_t_ method (cycle threshold) method. The uncoated scaffold cultured in basal media was used as an internal relative reference for the other scaffolds and media types. Each sample was a biological triplicate and plated out once each.

### Chorioallantoic Membrane assay

Descriptions are extrapolated from Marshall et al*.*^23^. All procedures were performed with prior received ethical approval from the University of Southampton Animal Welfare and Ethics Review Board and in accordance with the guidelines and regulations of the University of Southampton and those stated in the Animals (Scientific Procedures) Act 1986 and using the Animal Research: Reporting of In Vivo Experiments (ARRIVE) guidelines. However, a UK Personal Project License (PPL) was not required as the eggs were used only until embryonic day (ED) 14 when the experiment ended. Briefly, fertilised hens (*Gallus gallus domesticus*) eggs (Medeggs, Henry Stewart & Co., Lincolnshire) were incubated in a humidified (60%), warm (37 °C) incubator (Hatchmaster incubator, Brinsea, UK) at embryonic day (ED) 0. The eggs were incubated in a horizontal position, on a rotating pattern (1 h scheduled rotation), prior to albumin removal at ED 3 (Supplementary Figure 6). The minimum number of eggs required to determine a significant difference (*p* < 0.05) between groups was calculated with power 80%. Typically, an n = 6 for each condition (to allow predominantly for non-developing eggs) was used.

#### Scaffold preparation and implantation of materials

All PCL-TMA scaffolds (uncoated, ELP coated and/or PEA coated) were EO sterilised. The PEA scaffolds and ELP/PEA coated scaffolds were coated with FN (20 μg/mL) and BMP-2 (5 μg/mL) solutions immediately prior to use. Scaffolds were implanted at ED 7. Descriptions are extrapolated from Marshall et al.^23^. In brief, eggs were candled to check viability and a No. 10 scalpel blade was used to create a 0.5 cm by 0.5 cm window. The white inner shell membrane was peeled away and the materials placed onto the CAM. Parafilm was used to cover the window and labelled tape applied, parallel to sides of the egg, to hold the parafilm in place. The eggs were placed horizontally within an egg incubator at 37 °C and 60% humidity without rotation.

#### Analysis of results

Samples were harvested at ED 14 of incubation with methodology extrapolated from Marshall et al.^23^. Blinding of the assessor was performed. The window was opened digitally and with forceps to image the scaffold/CAM using a stemi 2000-c stereomicroscope (Zeiss, UK), for illustrative, recording purposes only. Quantification of angiogenesis was performed using the Chalkley eyepiece graticule scoring method. Three separate counts were made and the average score was calculated for each egg. Biocompatibility was assessed by counting live, viable and developed chicks and any dead/deformed chicks. Thereafter, the scaffold and 1 cm diameter of surrounding CAM tissue was collected and placed into 2 mL 4% PFA in a 24 well plate for 72 h at 4 °C followed by exchange of PFA for 70% ethanol. Processing of the scaffold/tissue, glycidyl methacrylate (GMA) resin embedding and subsequent histology followed. The chick was euthanised by an approved schedule 1 method at ED 14.

## Murine subcutaneous implantation study

### Mouse type and housing

All procedures were performed in accordance with University of Southampton institutional guidelines and regulations and with ethical approval from the Animal Welfare and Ethics Review Board (AWERB). The study was performed under PPL P96B16FBD, in accordance with the regulations in the Animals (Scientific Procedures) Act (ASPA) 1986 and using the Animal Research: Reporting of In Vivo Experiments (ARRIVE) guidelines. Training in all parts of the procedure including mouse handling, performing anaesthesia and surgery was provided and demonstration of competency was confirmed prior to the study as per the UK Home Office guidance and ASPA. Nine, adult (4 months old), male MF-1 wild type mice, bred on site and group housed in individually ventilated cages (IVCs) were used. Power/sample size calculations have determined that sample sizes of 4–6 animals per group using a p-value of 0.05 would give ≥ 90% power calculations. Mice had access to ad libitum standard pellets and water.

### PCL-TMA scaffold and collagen sponge with BMP-2 preparation for subcutaneous implantation

The PCL-TMA scaffolds were uncoated, ELP and/or PEA coated followed by EO sterilisation. The PEA coated and ELP/PEA coated scaffolds had FN and BMP-2 applied immediately prior to use. Three scaffolds were used per vacutainer therefore theoretically 6.6 µg FN and 1.6 µg BMP-2 could be adhered to each scaffold if coated equally. The positive control was a collagen sponge disc (4 mm diameter, 2 mm high) soaked with 5 µg of BMP-2 within 15 µL of InductOs® buffer solution (333.33 µg/mL) (Supplementary Table 1) immediately prior to use. Nine mice were used in total. An n = 6 coated scaffolds and n = 9 in the uncoated PCL-TMA scaffold and n = 9 in the collagen sponge/BMP-2 control groups were used. The three additional uncoated control and collagen sponge implants were to ensure the same number of implants (n = 4) per mouse and to ensure each mouse had a positive and negative scaffold/collagen sponge implanted with two coated scaffolds (Supplementary Figure 7).

### Subcutaneous implantation surgical procedure

General anaesthesia was induced with volatile Isoflurane (5%) and 100% oxygen (0.8 L/min flow rate) and this was continued for the maintenance of a surgical plane of anaesthesia at approximately 1.5–2.5% isoflurane. Once anaesthetised, Lubrithal eye gel was applied, ear marking performed for identification, the dorsum of the mouse was shaved and chlorhexidine/alcohol solution (Vetasept® Clear Spray) applied to the dorsum and allowed to dry. Buprenorphine (Buprecare® multidose, 0.05 mg/kg) was administered subcutaneously. Four dorsal 5 mm incisions were made (2 on each side over the shoulder and flank regions) and the skin elevated using blunt dissection, with a scaffold or collagen sponge/BMP-2 implanted into each subcutaneous pouch. The skin incisions were closed in a simple interrupted, horizontal mattress suture pattern with absorbable 5/0 PDS (Ethicon, USA), with the knots towards midline. After observed movement, the mouse was transferred to a heating box (Thermacage™, datesand group, UK) prewarmed to 30 °C until normal mouse activity resumed (eating, walking, grooming) at which point the mouse was returned to IVC housing.

### Micro-CT procedure and analysis of results

Micro-CT (µCT) was performed using a MILabs OI-CTUHXR preclinical imaging scanner (Utrecht, The Netherlands). The scaffolds/collagen sponges were scanned in vivo at weeks 2, 4, 6 and the excised materials only at week 8. General anaesthesia was induced using 5% isoflurane in an induction box, the mouse moved to the imaging bed and maintained at 1.5–2.5% isoflurane throughout imaging, with the oxygen flow rate constant at 0.8–1.0 L/minute. BioVet software connected to the Milabs scanner, permitted monitoring of respiratory activity (minimum 60 breaths per minute) and setting of the temperature of the scanning bed to 34 °C. Lubrithal eye gel was applied before imaging to prevent eye desiccation throughout the scan. Mice were imaged using 3 bed positions to µCT scan from the neck to the base of the tail, with a total scan time of approximately 15 min. Mice were recovered on the warm imaging bed followed by the heating box. After 8 weeks, to obtain higher resolution images; scaffolds were retrieved from the mice post-mortem and scanned in a specialised sample holder. A density phantom, as a reference for quantification of bone density, was scanned using the same parameters (Supplementary Table 2). μCT reconstructions were obtained via MILabs software (MILabs-Recon v. 11.00). Formation of bone was assessed using Imalytics Preclinical software v3.0 (Gremse-IT GmbH). A gauss filter of 0.5 and bone density threshold for analysis was set equal to the average result from the lower density bone phantom.

### Histology

#### GMA resin embedding and sectioning of samples

Tissue samples with scaffolds/collagen sponge were fixed in 4% PFA at 4 °C for up to 7 days and stored in 70% ethanol at 4 °C. The PCL-TMA scaffolds and collagen sponge/BMP-2 samples were placed in glass vials of ice-cold acetone containing 2 mM phenyl methyl sulphonyl fluoride and 20 mM iodoacetamide overnight at −20 °C. The solution was removed and acetone added for 15 min at RT, this was removed and methyl benzoate added for 15 min at RT. The methyl benzoate was removed and a solution of 5% methyl benzoate in glycerol methacrylate (GMA solution A) was added and incubated at 4 °C for 6 h total, with fresh solution added after 2 and 4 h. After 6 h, this solution was removed and embedding solution was made by adding 10 mL GMA solution A to 70 mg benzoyl peroxide and swirling until the powder dissolved. A 250 µL aliquot of GMA solution B was added and swirled by hand until the colour became off-yellow. The samples were placed into labelled plastic embedding tubes and the resin pipetted to completely fill each tube, with an ellipse at the top and the lid closed firmly. The samples were placed in the fridge at 4 °C with a weight on top to keep the lids tight to exclude any air and left for at least 48 h. The samples were transferred to a plastic box with silicone granules and stored at −20 °C. Samples were sectioned at 10 µm using a Reichert-Jung microtome with tungsten carbide Micron blade and floated in water onto glass slides. The slides were dried at RT until staining.

#### Histology staining

Staining was performed without prior rehydration of the sample sections and was performed with Alcian Blue and Sirius Red, Goldner’s Trichrome, Alizarin Red and Von Kossa stains followed by blotting to dry and mounting with dibutyl phthalate xylene (DPX) and a glass coverslip and allowed to dry. Histological samples were imaged using the Zeiss Axiovert 200 digital imaging system using bright field microscopy with the halogen bulb. Images were captured using the Axiovision 4.2 imaging software.

#### Alcian blue/Sirius red staining

Weigert’s Haematoxylin was applied for 10 min to stain the cell nuclei. Excess stain was removed by rinsing 3 times in acid/alcohol (5% HCl/70% ethanol) followed by dripping on water for 5 min. 0.5% Alcian blue 8GX in 1% acetic acid was applied for 10 min. Slides were covered in 1% molybdophosphoric acid for 10 min, followed by rinsing in water prior to staining with 1% Picrosirius Red (Sirius Red) for 1 h and excess stain was rinsed off with water.

##### Goldner’s Trichrome staining

The method followed was identical to Alcian blue/Sirius Red staining to the point of acid/alcohol use and rinsing with water for 5 min. Ponceau Acid Fuchsin/Azophloxin was applied for 5 min followed by a 15 s wash with 1% acetic acid dripped onto the slide. Phosphomolybdic acid/Orange G was applied for 20 min followed by another 15 s wash with 1% acetic acid. Light Green was applied for 5 min followed by the third 15 s wash with 1% acetic acid.

##### Von Kossa staining

The slides were incubated with 1% silver nitrate under UV light for 20 min. Slides were washed using water and incubated with 2.5% sodium thiosulfate for 8 min, washed with water and counterstained with Alcian blue for 1 min. The slides were washed in water and van Gieson’s stain was applied for 5 min.

##### Alizarin red/Light green staining

Alizarin red S (40 mM) stain solution was applied for 2 min. The slide was blotted on paper towel and light green stain applied for 2 min as a counterstain. This was blotted off and acetone followed by acetone/Histoclear (50:50, (v/v)) followed by Histoclear were applied for 30 s each and the excess allowed to evaporate prior to mounting.

### Statistical analysis

AlamarBlue™ and biochemistry results were established with biological triplicates, with triplicate readings taken from each sample and the average of the triplicate readings used for statistical analysis. Molecular experiments were run using biological triplicates with one reading taken from each sample. Statistical analysis was performed comparing uncoated scaffolds to each coated type and in basal and osteogenic conditions where applicable using a 2-way ANOVA with Dunnett’s multiple comparisons test. Statistical analysis of the molecular results used the ΔCT values, while the graphs displayed show the 2^-ΔΔCT^ values to display fold change in relation to the uncoated scaffolds cultured in basal media. Molecular results data was analysed using a 2-way ANOVA with Dunnett’s multiple comparisons test. For the CAM assay, data was analysed using a one-way ANOVA with Dunnett’s multiple comparisons test. Set up used n = 6 eggs per condition, but only the mean and S.D. of the Chalkley score results from formed/viable eggs or those able to be counted were included in statistical analysis. In the murine subcutaneous implantation study, data from replicates were analysed using a one-way ANOVA with Dunnett’s multiple comparisons test. Analysis and graphical presentation were performed using GraphPad Prism 9, version 9.2.0. P values < 0.05 were considered significant. Graphical representation of significance as follows: ns is no significant difference, *p < 0.05, **p < 0.01, ***p < 0.001, ****p < 0.0001. All data presented as mean and standard deviation (S.D).

## Results

### Characterisation of the PCL-TMA material and analysis of material properties

**Fig. 1:**
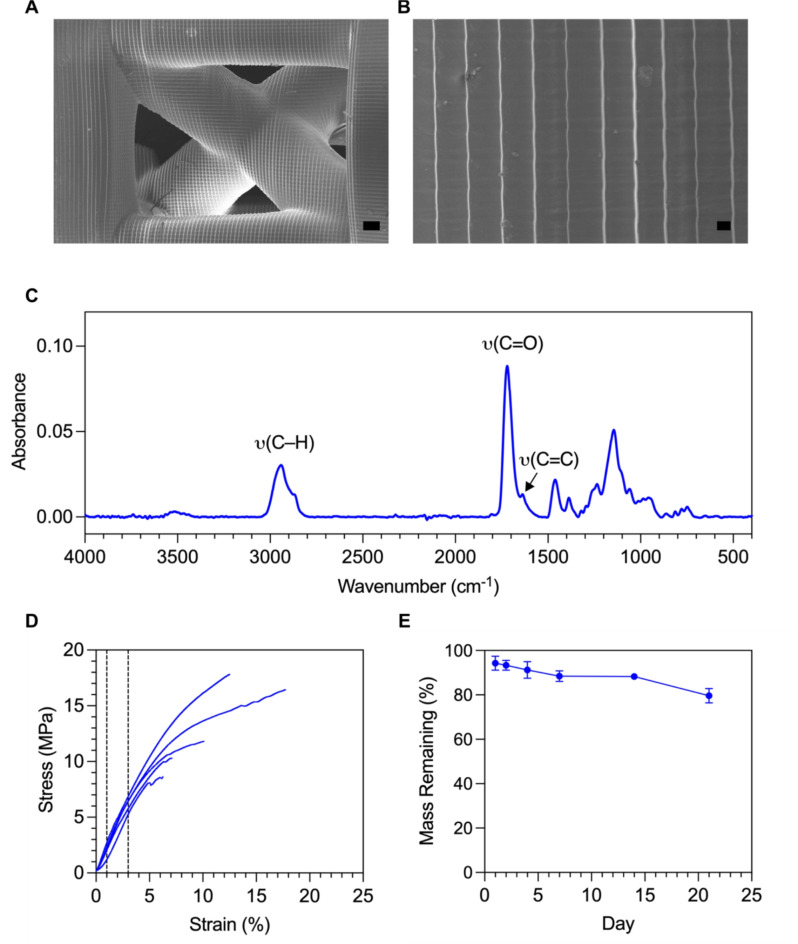
Properties of the PCL-TMA material. SEM micrographs of the PCL-TMA unit cell scaffolds. (**A**) shows the central octet-truss unit cell structure (scale bar = 200 µm) and (**B**) shows a zoomed image of the layer lines, which shows excellent agreement with the sliced layer height of 50 µm (scale bar = 20 µm). (**C**) ATR-FTIR of the 3D printed PCL-TMA unit cells. Key vibrational modes are annotated on the spectra. (**D**) Compressive testing of the 3D printed PCL-TMA unit cells (n = 5). The effective elastic modulus was determined between 1 – 3% strain. (**E**) Accelerated degradation study of the 3D printed PCL-TMA unit cell scaffolds (n = 3) in 2 M NaOH at 37 °C.

The PCL-TMA materials were synthesised using modified literature procedures, with the main difference being an additional silica purification step to improve the purity of the material^8–10^. We designed a scaffold based on the unit cell of the octet-truss scaffolds in our previous work^7^. We characterised the surfaces of the unit cell scaffolds, with SEM showing layer lines which are in excellent agreement with the slicing thickness of the model (Fig. 1A and B). We characterised the PCL-TMA materials using ^1^H NMR, which showed a degree of functionalization > 95% (Supplementary Figure 8). As consistent bioactive coatings on the PCL-TMA materials are a priority, we rationalised that using these unit cell scaffolds would allow for coating optimisation using identical geometries and strut sizes as those which would be used in larger animal models. The unit cell scaffolds were printed using a Prusa SL1S 3D Printer as described in section "3D Printing of PCL-trimethacrylate" and FTIR showed almost complete conversion of the methacrylate groups on the surface of the scaffolds (Fig. 1C). We investigated the mechanical properties of the unit cell scaffolds using compressive testing (Fig. 1D, Supplementary Table 3) which demonstrated an effective elastic modulus of 203.7 ± 11 MPa. The scaffolds also demonstrated strains approximately two-fold the ultimate compressive strain of cancellous bone. Importantly, in accelerated degradation tests (2 M NaOH, 37 °C) we observed degradation of the unit cell scaffolds (Fig. 1E), validating their potential to degrade via hydrolysis.

In this current study, the ELP coated PCL-TMA scaffolds displayed increased brittleness following coating with a mineralisation solution, limiting this combination for lower limb bone repair. The ELP coating uses a *bis*(isocyanate) crosslinker as a functional handle to attach the ELP to the PCL-TMA. We know from the NMR and FTIR analysis that some hydroxyl groups from the PCL-TMA remain (approx. 5% from ^1^H NMR). The *bis*(isocyanate) crosslinker will react with these hydroxyl groups, which increases the cross-linking density and reduces the distance between cross-linking points, ultimately making the material more brittle. The ELP coating itself was compared to ELP/mineralised coating on polyamide (PA12) in an optimisation experiment, to determine that in osteogenic conditions, the ELP coating held osteogenic functionality properties (Supplementary Figure 9). Therefore, the ELP coating was assessed without being exogenously mineralised and with concurrent PEA/FN/BMP-2 coating of the PCL-TMA scaffold material.

### In vitro cell viability and osteogenic differentiation on PCL-TMA scaffolds

#### The PCL-TMA material and bioactive coatings were cytocompatible with HBMSC attachment and growth over 14 days

**Fig. 2:**
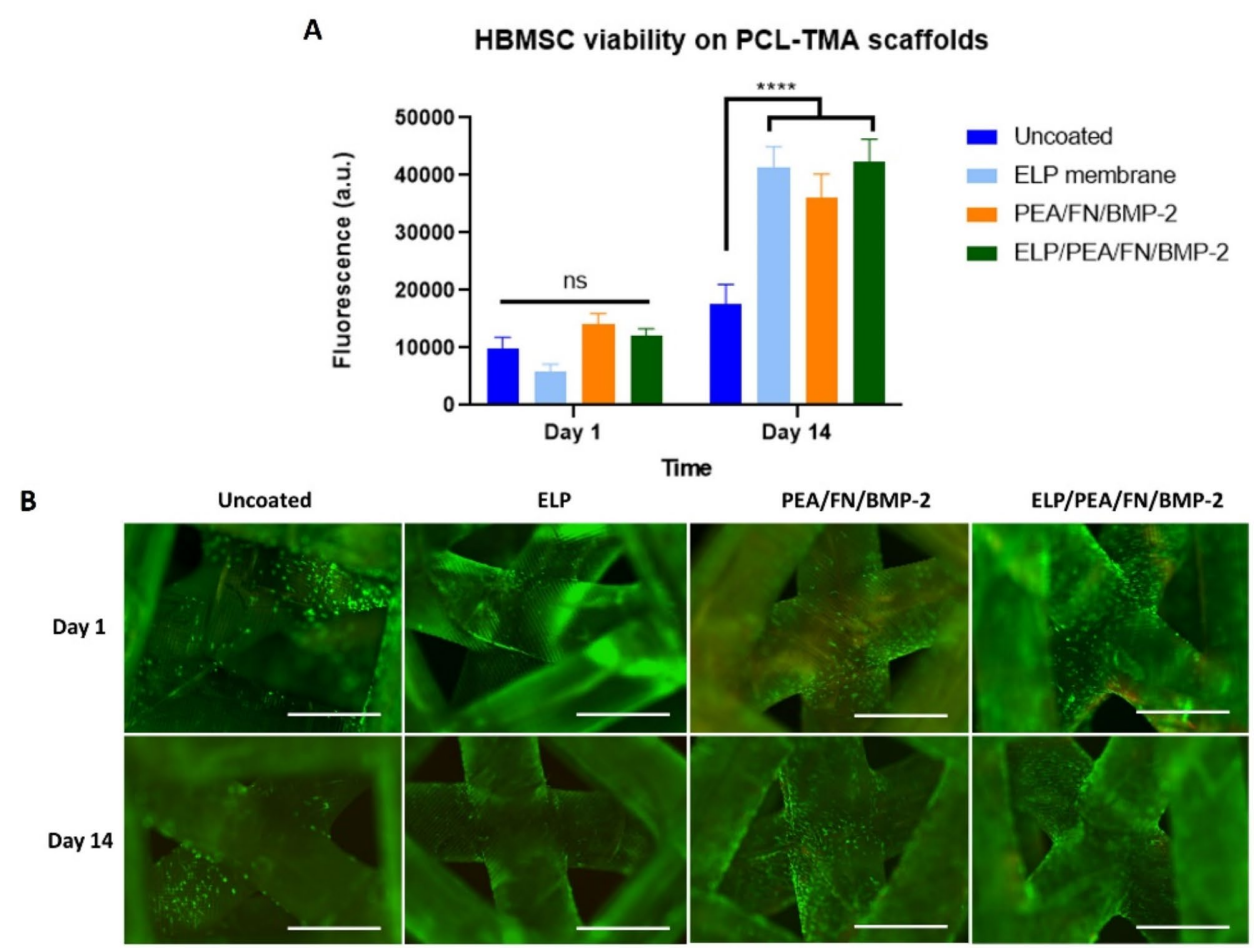
HBMSC viability and live (green)/dead (red) cell labelling on uncoated, ELP, PEA/FN/BMP-2 and ELP/PEA/FN/BMP-2 coated PCL-TMA scaffolds. (**A**) alamarBlue™ HS fluorescence results of ELP, PEA/FN/BMP-2 and ELP/PEA/FN/BMP-2 coated PCL-TMA scaffolds 2-way ANOVA with Dunnett’s multiple comparisons test, n = 3, mean and S.D. shown, ns; non-significant, ****p < 0.001. (**B**) The cell number and location of cells adhered to the scaffold initially at day 1 and subsequent increase in cell coverage at day 14, as seen by fluorescent labelling of cells. Scale bar 1 mm.

Cell number was measured using alamarBlue™ HS reduction as a surrogate marker (Supplementary Figure 10). Preliminary studies confirmed the PEA/FN/BMP-2 coating was cytocompatible on PCL-TMA scaffolds and the PEA/FN/BMP-2 bioactive coating was observed to increase cell adhesion, as determined by enhanced fluorescence values obtained at day 1, although this did not reach significance (Supplementary Figure 11). The ELP, PEA/FN/BMP-2 and ELP/PEA/FN/BMP-2 coatings were observed to be cytocompatible with an increase in alamarBlue™ HS fluorescence results (Fig. 2A) and cell number at day 14 (*p* < 0.0001), as observed by fluorescent staining of the cells (Fig. 2B). The PEA/FN/BMP-2 coating on the PCL-TMA scaffold, or in conjunction with the ELP coating, were observed to enhance cell adhesion to the PCL-TMA scaffold material after 24 h as evidenced by increased alamarBlue™ HS fluorescence, however this was not significant.

#### Osteogenic differentiation of HBMSCs in response to ELP coated, PEA/FN/BMP-2 and ELP/PEA/FN/BMP-2 coatings on 3D scaffolds

**Fig. 3:**
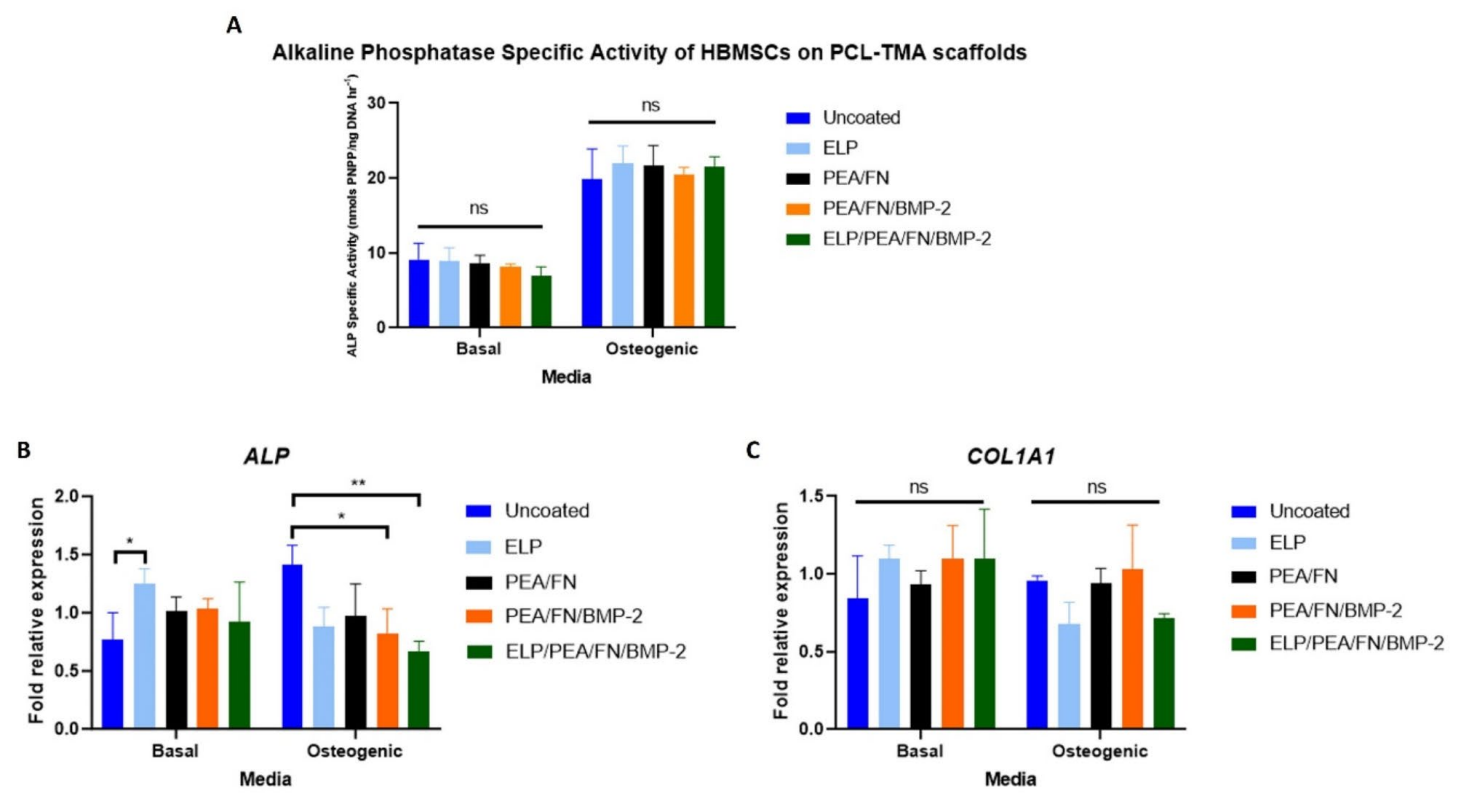
Assessment of HBMSC differentiation on coated 3D scaffold materials. (**A**) ALP specific activity of HBMSCs on ELP, PEA/FN/BMP-2 and ELP/PEA/FN/BMP-2 coated PCL-TMA scaffolds at day 7. (**B**) ALP gene expression of HBMSCs on PCL-TMA scaffolds at day 7 (**C**) Collagen1A1 gene expression of HBMSCs on PCL-TMA scaffolds at day 7 was not significantly greater than uncoated polyamide for any of the coatings in basal or osteogenic media conditions. 2-way ANOVA with Dunnett’s multiple comparisons test, n = 3, mean and S.D. shown, ns; non-significant, *p < 0.05, **p < 0.01.

The ELP coated, PEA/FN/BMP-2 coated, or combined coating did not significantly increase ALP activity of HBMSCs in basal or osteogenic culture conditions compared to HBMSCs cultured on uncoated PCL-TMA scaffolds when 100 ng/ml BMP-2 was used in the coating solution (Fig. 3A). Due to these unexpected ALP specific activity results, optimisation and confirmatory experiments were performed. During the confirmatory experiments, the PEA/FN/BMP-2 coating using 100 ng/mL BMP-2 exhibited minimal ALP staining in C2C12 cells, whereas a significantly positive response was observed with a 5 µg/mL BMP-2 coating solution, indicating the BMP-2 was bound to the well/scaffold by the PEA/FN (Supplementary Figures 2 and 3). Furthermore, it was found that the pH of the diluent used affected BMP-2 activity, while manufacturer did not (Supplementary Figures 2, 3 and 4). In addition, specific ALP activity was increased by HBMSCs cultured on polyamide scaffolds coated with 5 µg/mL compared to 100 ng/mL BMP-2 (Supplementary Figure 12). Therefore, these experiments confirmed that the concentration of BMP-2 to be used in vivo i.e. 5 µg/mL would more likely produce mineralisation due to the encouraging ALP results and that 100 ng/mL was not sufficient to stimulate a response from the HBMSCs. Further, it was noted, a neutral diluent of PBS should be used for the BMP-2.

The osteogenic gene expression of *ALP* and *COL1A1* were assessed for the ELP coating, PEA/FN/BMP-2 coating (at 100 ng/ml BMP-2) and combined coatings at day 14 of culture in basal and osteogenic conditions. *ALP* gene expression was significantly greater for ELP coating than uncoated PCL-TMA (*p* < 0.05) in basal culture conditions. In contrast, PEA/FN/BMP-2 and ELP/PEA/FN/BMP-2 coatings displayed significantly reduced *ALP* gene expression under osteogenic culture conditions (Fig. 3B). *COL1A1* gene expression was not significantly different under any bioactive coatings examined in comparison to uncoated PCL-TMA following culture in basal or osteogenic media conditions (Fig. 3C). This was not repeated at 5 µg/ml due to the volumes of BMP-2 required to coat the multiple scaffolds and subsequently lost after coating the scaffolds.

The hypothesis that a dual coating of ELP/PEA/FN/BMP-2 would be synergistic in vivo compared to each coating alone was examined. The assumption was that HBMSCs would adhere to the scaffolds initially, due to the presence of FN and BMP-2 followed by enhanced mineralised matrix in response to the ELP/BMP-2. Therefore, the ELP, PEA/FN/BMP-2 and ELP/PEA/FN/BMP-2 coating options on PCL-TMA scaffolds were assessed in the CAM assay.

### The PCL-TMA material and bioactive scaffolds were biocompatible following evaluation on the CAM assay

**Fig. 4:**
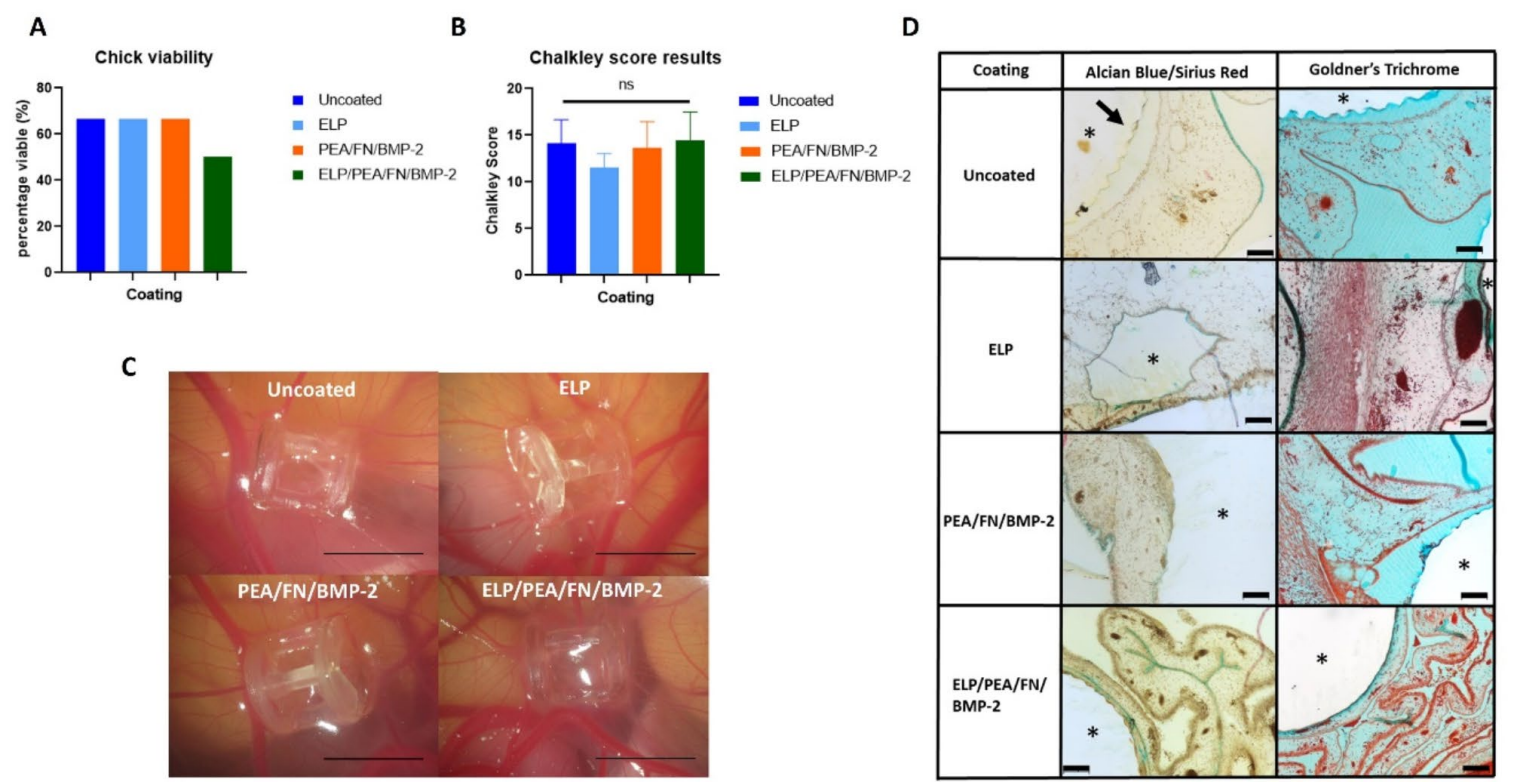
CAM assay viability and Chalkley score results for PCL-TMA scaffolds. (**A**) Chick viability was suboptimal due to poor chick development, n = 6. (**B**) There was no significant difference in Chalkley score between uncoated PCL-TMA and the coated scaffolds, (uncoated n = 4, ELP n = 4, PEA/FN/BMP-2 n = 4, ELP/PEA/FN/BMP-2 n = 3), ns; non-significant. One-way ANOVA with Dunnett’s multiple comparisons test was used for statistical analysis, mean and S.D. shown. (**C**) Photographs of representative uncoated PCL-TMA and ELP coating, PEA/FN/BMP-2 and ELP/PEA/FN/BMP-2 coated PCL-TMA scaffolds on the CAM. Scale bar 5 mm. (**D**) Histological staining of PCL-TMA scaffolds surrounded by CAM tissue. The PCL-TMA scaffold material did not support sectioning, with fragments remaining (arrow), but tissue around the prior scaffold (*) could be determined. Scale bar 100 μm.

PCL-TMA scaffolds with ELP, PEA/FN/BMP-2 and ELP/PEA/FN/BMP-2 coatings were analysed for biocompatibility and angiogenic support using the CAM assay. All three coatings on PCL-TMA showed excellent biocompatibility and the ability to support angiogenesis, although viability scores were reduced due to undeveloped chicks (Fig. 4A). Analysis demonstrated no significant difference in Chalkley score between uncoated PCL-TMA scaffolds and PCL-TMA scaffolds with coatings, when analysed by a blinded observer (Fig. 4B). The scaffolds were all noted to integrate with the CAM with blood vessels clearly visible, surrounding each scaffold, with no gross thickening or disruption of the CAM tissue due to the scaffolds (Fig. 4C). The PCL-TMA scaffolds were noted to be surrounded by CAM tissue, blood vessels and host cells from the CAM evidenced following Alcian blue and Sirius red or Goldner’s trichrome staining (Fig. 4D). The brittle nature of the PCL-TMA led to the polymer material shearing on sectioning, despite being embedded in hard GMA resin. Overall, the various bioactive coatings were biocompatible when combined with the PCL-TMA material, with no indication of an inflammatory response observed.

### Bone formation analysis on the PCL-TMA scaffolds compared to collagen sponge with BMP-2 using the mouse subcutaneous implantation assay.

#### µCT analysis results of uncoated scaffolds compared to coated PCL-TMA scaffolds and collagen sponge/BMP-2

**Fig. 5:**
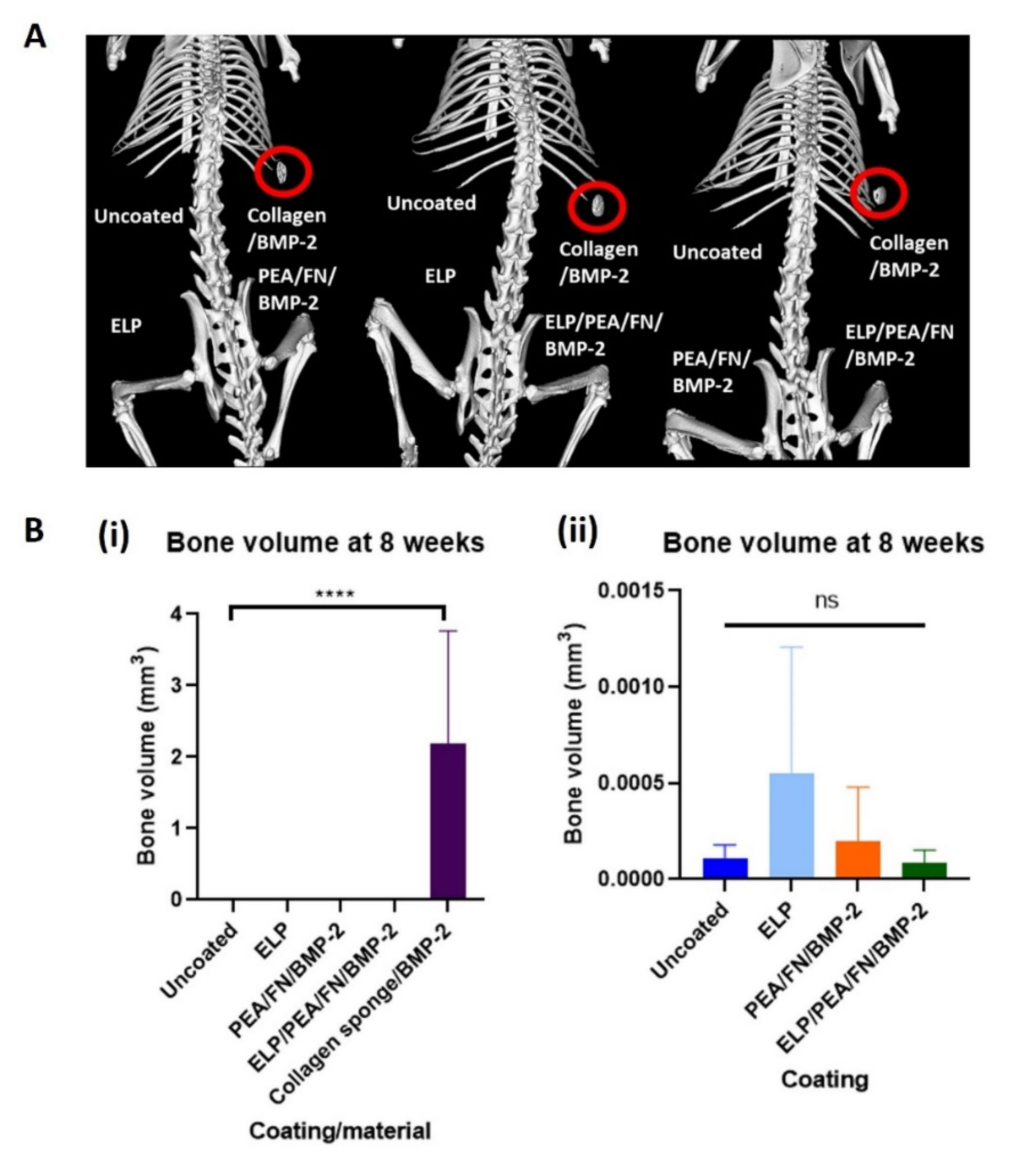
µCT results of the murine subcutaneous implantation study. (**A**) Representative µCT images with no bone formation observed in uncoated, PEA/FN/BMP-2, ELP, or ELP/PEA/FN/BMP-2 coated PCL-TMA scaffolds, however the collagen sponge with 5 µg of BMP-2 showed mineralisation in all mice (within red circles) at week 6. (**B**) Quantification of bone volume formed in the mouse subcutaneous implant model using PCL-TMA scaffolds and collagen sponge/BMP-2. One-way ANOVA with Dunnett’s multiple comparisons test, ns; non-significant, ****p < 0.0001. N = 9 uncoated scaffolds, n = 9 collagen sponge, n = 6 PEA/FN/BMP-2 and n = 6 ELP/PEA/FN/BMP-2 coated scaffolds, mean and S.D. shown.

The mouse subcutaneous implantation study demonstrated that the PCL-TMA scaffolds displayed no detectable mineralisation at week 2, 4 or 6 scans in vivo. In contrast, extensive mineralisation was observed in the collagen sponge with 5 µg BMP-2 from week 2 onwards, clearly seen in representative week 6 images, as a mineralised disc (Fig. 5A). The excised samples from the mice were scanned ex vivo at week 8 and no significant mineral was detected in response to either of the ELP or PEA/FN/BMP-2 coatings, or when applied concurrently. The collagen sponge displayed significant mineralisation (*p* < 0.0001) due to the response of the adipose and subcutaneous stromal cells to the BMP-2, when quantified at week 8, compared to the uncoated PCL scaffolds (Fig. 5B (i)). There was no significant difference between the negligible quantities of bone formed on the coated scaffolds compared to the uncoated PCL-TMA scaffolds (Fig. 5B (ii)).

#### Histological analysis of the subcutaneous PCL-TMA scaffolds and positive control collagen sponge/BMP-2

**Fig. 6:**
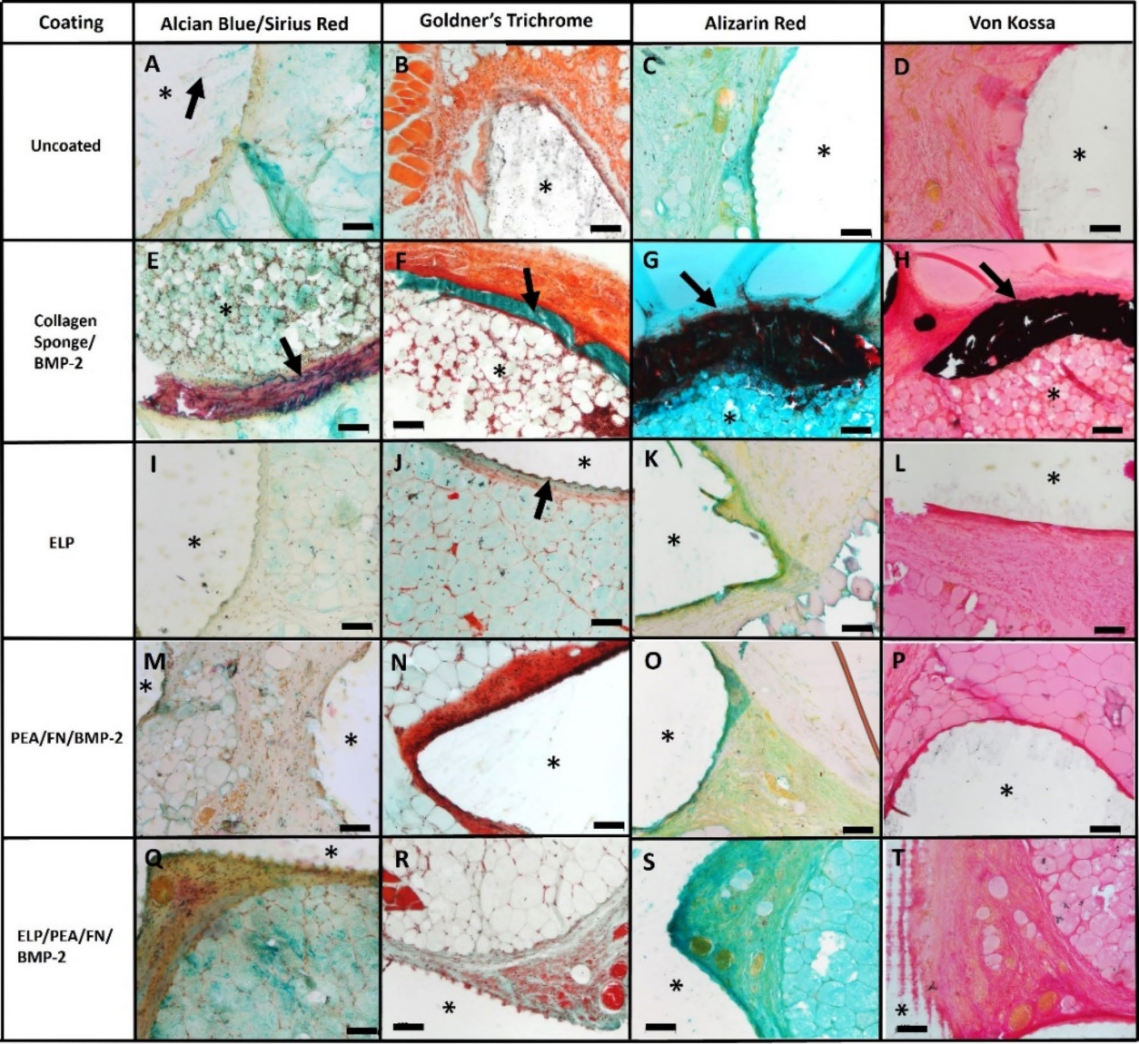
Alcian blue and Sirius red, Goldner’s trichrome, Alizarin red and Von Kossa staining of PCL-TMA scaffolds and collagen sponge/BMP-2. The scaffolds were not amenable to sectioning; however, the surrounding tissue remained. (*) PCL-TMA scaffold area or collagen sponge/BMP-2. (**A**) Shards of PCL-TMA material (black arrow) remain in the section. (**B**) Vivid red staining muscle was seen but no bone formation. (**C** and **D**) No bone formation was found on the uncoated scaffold. (**E**–**H**) Only the collagen sponge displayed marked mineralisation and bone formation around the periphery (arrows). (**I**–**T**) No bone formation was seen on the ELP, PEA/FN/BMP-2 or ELP/PEA/FN/BMP-2 coated scaffolds, with the ridges of the scaffold material seen surrounded by tissue (J arrow). Scale bar 100 μm.

Histological analysis of the PCL-TMA bioactive scaffolds following subcutaneous implant studies showed discrete shards of fragmented PCL-TMA material from the periphery of each PCL-TMA scaffold (coated and uncoated) (example Fig. 6A). The surrounding tissue was integrated with the scaffold as evidenced by the scaffold ridges within the tissue (example Fig. 6J). Alcian blue and Sirius red staining indicated the presence of proteoglycans within the surrounding tissue and a discrete red rim of bone tissue staining at the perimeter of the collagen sponge with BMP-2 (Fig. 6F). Goldner’s trichrome stain demonstrated collagenous bone matrix (green stain) in the collagen sponge/BMP-2 group (Fig. 6F), while the muscle tissue was stained vivid red in the uncoated scaffold example (Fig. 6B). Intensely red staining collagenous tissue was observed to surround the collagen sponge and the PEA/FN/BMP-2 coated scaffold (Figs. 6F and N). Alcian blue and Sirius red staining of collagen sponge demonstrated a rim of collagen-rich tissue matrix around the construct (Fig. 6E). Alizarin red staining produced a marked dark red stain around the collagen sponge with BMP-2, indicative of mineralisation as seen on µCT analysis (Fig. 6G). No detectable bone formation was seen on histological examination of the coated or uncoated PCL-TMA scaffolds (Fig. 6A–D, I–T). Von Kossa staining was comparable in location to the alizarin red, confirming the presence of mineralisation around the periphery of the collagen sponge/BMP-2 construct only (Fig. 6H). The perimeter of all PCL-TMA scaffolds examined showed no positive black staining, confirming an absence of bone formation (Fig. 6D, P, L, T).

## Discussion

The current study set out to examine the potential of bioactive 3D-printed octet-truss PCL-TMA scaffolds for bone formation augmentation. The in vitro studies examined cytocompatibility, the ability to induce ALP and mineral production and to enhance osteogenic gene expression within HBMSCs. All of the bioactive coatings examined showed no signs of cytotoxicity after 14 days and viable cells were visualised with live and dead cell labelling. Proliferative activity over time e.g. day 1, 3, 5, 7, etc. was not performed as the scaffolds were used for live/dead staining after the fluorescence readings were taken. Further, it was not practical to assess multiple timepoints due to the cell seeding method and HBMSC numbers used, or financially viable to coat the number of scaffolds which would be needed for each material and coating type. CAM assay analysis demonstrated biocompatibility of the PCL-TMA scaffold material and bioactive coatings with no effect on angiogenesis or chick development. The subcutaneous implantation study revealed marked bone formation around the collagen sponge with BMP-2, while there was no bone formation on any of the bioactive coated PCL-TMA scaffolds.

With regard to the scaffold material properties, whilst an increase in the degree of functionalisation gives the material favourable compressive properties, it also affects the degradation rate. The number of cross-linking points and distance between cross-linking points will dictate the rate of degradation through i) the number of ester bonds to be hydrolysed and ii) the rate of swelling in water of the material which will dictate the mechanism of degradation (i.e., bulk erosion or surface erosion). In an ideal scenario, a “goldilocks” material would degrade after load-supporting, mature bone has been regenerated. In practice, engineering this degradation time just based on hydrolysis of the material is difficult because the local pH, surface area of the scaffold, and effects of enzymatic degradation which is linked to inflammation needs to be considered. Nonetheless, we (vide infra) and others^8–10^ have shown in accelerated degradation studies that these materials degrade via base catalysed hydrolysis. The degradation of PCL-TMA via the ester groups likely generates a combination of small PCL-TMA oligomers, oligo(caprolactone) oligomers and poly(methacrylic acid) oligomers. The low molecular weight of these degradation products and the porous geometry of the scaffolds enables the degradation products to be efficiently removed in vivo by a multitude of factors (e.g., hydrolytic, enzymatic, mechanical, immune related).

It was found that the exogenously mineralised ELP coating altered the PCL-TMA scaffold material and therefore, a contingency plan to adjust the coating method followed by further experiments to ensure the efficacy of the ELP coating alone, without exogenous mineralisation, were essential prior to further experiments such as those in vivo. This was an important aspect of optimising the material and coatings to be safe, practical and robust with the view towards and ultimate goal of clinical application in human healthcare. It was found to be important to aim to continue with protocols throughout the project to allow previous work to remain valid and not become obsolete if major alterations were to be made, to ensure consistency from bench to bedside.

Several factors are known to affect cell differentiation including: i) material stiffness, ii) hydrophobicity, iii) the concentration, configuration and subsequent activity of growth factor due to the method of application and, iv) the culture time may impact on cell differentiation. The ALP specific activity results were not significantly different from uncoated scaffolds in basal media conditions for the PEA/FN/BMP-2 coating, with the bound BMP-2 unable to induce significant osteogenic differentiation of cells. It has been shown that less than 10% of the bound BMP-2 is released over a 14-day period, thus to explain the in vitro and in vivo findings, if only a small quantity is bound due to the relatively low surface area of the PCL-TMA material or possible hydrophobicity impacting on PEA coating uniformity, this may have led to the PEA/FN/BMP-2 coating being less efficacious than in reported studies^18^. Therefore, based on this finding it was difficult to make a firm conclusion given that the in vitro environment with encouraging results differs from the in vivo environment, limiting assessment of the coating capabilities. Interestingly, the PEA/FN/BMP-2 coating using 100 ng/mL BMP-2 exhibited minimal ALP staining in C2C12 cells, whereas a significantly positive response was observed with a 5 µg/mL BMP-2 coating solution on tissue culture plastic and PCL circular scaffolds, indicating the BMP-2 was bound to the well/scaffold by the PEA/FN. However, this PCL scaffold was of a different material type, hydrophobicity, and surface area to the PCL-TMA scaffolds and thus may permit greater PEA/FN/BMP-2 adhesion capability, highlighting the importance of optimisation of the underlying scaffold material.

Different species have differing sensitivity to BMPs, with human osteoblasts requiring dexamethasone with BMP-2, 4 or 7 to increase ALP activity compared to murine osteoblasts^24–26^. This effect was potentially observed in the current study, when HBMSCs were cultured on the PEA/FN/BMP-2 (5 μg/mL) coated polyamide scaffolds in osteogenic media. This higher concentration of 5 μg/mL BMP-2 was used in the CAM assay and murine in vivo studies, however, in vitro cell culture experiments do not always replicate in vivo cell differentiation^27^. Interestingly, the findings at 5 μg/mL in basal conditions in vitro may mirror our in vivo findings in a heterotopic site. However, the ability of the low mass of BMP-2 bound to scaffolds for prolonged periods of time to induce osteogenic differentiation of HBMSCs in osteogenic conditions in vitro was encouraging. In comparison to the milligram quantities used clinically in people, microgram quantities of BMP-2 would be welcomed to reduce the extensively known and publicised side-effects. This indicates potential for osteogenesis in an osteogenic in vivo environment, therefore using a bone defect model may be more appropriate.

As detailed in the supplementary information, an initial coating was undertaken using acidic buffer solution (pH 4.5) to dilute the InductOs® rhBMP-2, as pH was thought to be inconsequential on the success of the coating method or cell differentiation. However, the current studies indicate the importance of the pH employed. It is reported that InductOs® BMP-2 activity is pH dependent and aggregation occurs at pH 6.5, hence in order to prevent this, the buffer was established at pH 4.5^28^. However, when testing bioavailability and activity of BMP-2 on C2C12 cells diluted in the acidic buffer, BMP-2 was found to be either inactive or unbound to the FN. A neutral or basic environment can destabilize the disulfide bonds, damaging the dimer configuration and inactivating the BMP, however InductOs® BMP-2 generated a positive ALP staining in the neutral buffer at a higher concentration of 5 μg/mL^29^.

The difference in ALP gene expression when the ELP coating was applied alone compared to application of PEA/FN/BMP-2 on top of the ELP coating, may result in the PEA/FN/BMP-2 coating prohibiting the action of the ELP coating. It is known that gene expression does not always relate to the production of matrix or protein at the scaffold surface or implant/bone interface, therefore the higher ALP gene expression in the ELP coating only group did not appear to translate to higher ALP protein formed in ALP specific activity assessment^30^. Further, the gene expression analysis timings should be selected for the suspected peak activity e.g., *ALP* at day 7–14 and *COL1A1* at day 21 onwards to mature collagen matrix maturation. However, it was not practical to test different genes at different time points in this study due to the numbers of scaffolds and volumes of coating solutions which would be required. The ELP coating on polyamide scaffolds was found to have the potential to be mineralised by HBMSCs when exposed to a mineralising media environment visualized by alizarin red staining. Thus, the PCL-TMA scaffolds with ELP coating and/or PEA/FN/BMP-2 coatings were found to be cytocompatible and support osteogenic differentiation and mineralisation of the microenvironment, therefore these bioactive coating options were further assessed in vivo. The CAM assay provided an intermediary step with results for the uncoated PCL-TMA, ELP coating, PEA/FN/BMP-2 coating and the combined ELP/PEA/FN/BMP-2 coatings demonstrating excellent biocompatibility and angiogenic response using accepted angiogenic quantitation methodology^31–34^.

The mouse subcutaneous implantation model allowed the first stage of scaffold assessment in a rodent preclinical model and confirmed the biocompatibility of the PCL-TMA scaffold and coatings. The study also confirmed the inability to mineralise in a heterotopic site, compared to the commercially available and clinically applied collagen sponge with BMP-2. However, a limitation is that this model does not provide the cells from the bone niche which may be responsive to these material coatings. Further this model does not mirror the clinical application we are aiming for, compared to use of a small animal or large animal model. Limitations also exist due to the species differences as previously mentioned. Bone did not form on the scaffold constructs, whereas collagen sponge with BMP-2 displayed significant bone formation, highlighting the need for consideration of effective growth factor mass, as the mass of BMP-2 (5 µg) within the collagen sponge was likely much greater than that attached to the PCL-TMA scaffolds, and the need for appropriate animal model use. The objective was to determine the optimal osteogenic coating on the PCL-TMA scaffold; however, the results did not determine one optimal coating from the ELP and/or PEA/FN/BMP-2 options using the subcutaneous implantation model, despite a sufficient period for mineralisation to occur. The PEA/FN/BMP-2 coating has been used successfully in osseous defect sites, with as little as 15 ng of BMP-2 in the murine radial defect model^18^. An ELISA using BMP-2 solutions to determine the mass of BMP-2 adhered to the PEA/FN coated PCL-TMA scaffolds was not performed due to difficulties attributed to aggregation of the protein following freezing at −20 °C^35^. The BMP-2 concentration released was not measured either, as the purpose of the coating is to adhere the BMP-2 to FN which has been shown to maintain 90% of the adsorbed BMP-2 after 14 days^18^. It may be that the quantity of BMP-2 bound from a 5 µg/mL solution to the PCL-TMA material may be less than required for a response by the subcutaneous tissues given the ‘critical mass’ required to induce mineralisation in rodents has been indicated to be around 1 µg of BMP-2, from review of the published effective and ineffective doses used^36^. A challenge is the lack of response that human skeletal cell populations display, assessed via ALP activity, to BMP-2 compared to rodent derived cells which can lead to discrepancy between rodent models and human clinical data^26^. Reporting the concentration and volume of growth factor use in animal models and details of material size and surface area allows more accurate comparison between animal studies, especially subcutaneous implant studies, as these vary as reported by Gothard et al.^37^. Consideration for the coating method and mass of growth factor delivered, often limited by the surface area of the scaffold, possibly limit the efficacy of this PEA/FN/BMP-2 coating when used in this model. Therefore, an osseous defect site may enhance the response to BMP-2 presentation by the scaffold or if the scaffold is optimised to increase the BMP-2 binding by altering the surface area and hydrophobicity for PEA/FN coating. The theoretical action of the ELP coating entails its ability to mineralise by sequestering surrounding calcium and phosphate ions, however, it is possible that the ELP coating could not access mineral to ‘grow’ on the surface of the ELP coating in the subcutaneous space. Therefore, application in an osseous defect model may offer a more suitable model and environment to test this bioactive coating. Future studies will use optimised PCL-TMA as it was found to be a hard material which may shatter when used as a press-fit into bone defect sites to improve useability and to apply the coatings in an osteogenic environment with skeletal cellular populations such as periosteal cells, endosteal cells and adipose cells within a femur defect model as these cellular populations may be more responsive to low dose BMP-2 delivery or mineralisation of the ELP coating.

## Conclusion

The PCL-TMA material and bioactive coatings were cytocompatible and biocompatible, with promising in vitro results. However, the ELP, PEA/FN/BMP-2 and ELP/PEA/FN/BMP-2 coatings were ineffective at inducing bone formation in the subcutaneous implantation model of heterotopic bone formation. These results indicate the importance of the selection of an appropriate model for material/growth factor assessment and bone formation mechanism determination. The absence of bone formation on the PCL-TMA scaffolds, in vivo, was potentially a consequence of the method of action of the applied coatings, the surface area of the scaffold construct for BMP-2 binding and the necessity of an appropriate in vivo environment to facilitate skeletal cell ingress. An osseous site defect may be more appropriate to test the ELP, PEA/FN/BMP-2 and ELP/PEA/FN/BMP-2 coatings if a more vascular, osteogenic site is required to initiate or enhance the response of skeletal cells to these coatings. Therefore, prior to discounting these coating materials for clinical translation, the use of an orthotopic rodent bone defect model would be warranted. The potential clinical regulatory pathway for these material scaffolds would involve further in vivo validation, including a large animal bone defect study to scale-up the potential bone defect volume and the mass the construct can carry, ISO 10993 testing of the scaffold and coatings as a medical device, and clinical trials using the final, custom-made, 3D-printed, sterile, acellular, coated scaffold to implant into human patients to heal significant bone tissue deficits.

## Electronic supplementary material

Below is the link to the electronic supplementary material.

Supplementary Material 1

## Data Availability

All data associated with this study are presented in the paper or the Supplementary Materials. All raw data is available on reasonable request from the corresponding authors.
